# Health-related quality of life among nursing home residents compared to community-dwelling and general older populations: influence of sociodemographic determinants and common chronic diseases

**DOI:** 10.1007/s10354-025-01111-w

**Published:** 2025-10-07

**Authors:** Matei Capatu, Christina Fastl, Daliah Kubik, Andrea Kapounek, Markus Mattersberger, Harald Sidak, Christoph Gisinger, Thomas E. Dorner

**Affiliations:** 1 Haus der Barmherzigkeit, Academy of Ageing Research, Seeböckgasse 30a, 1160 Vienna, Austria; 2 Medical University Vienna, Centre for Public Health, Department of Social and Preventive Medicine, Kinderspitalgasse 15/I, 1090 Vienna, Austria

**Keywords:** Quality of life, Quality indicators, healthcare, Geriatrics, Nursing homes, Aged

## Abstract

Health-related quality of life (QoL) is a key indicator of health and care quality in geriatric nursing homes. We assessed QoL in 190 residents of three nursing homes with questionnaires developed by the World Health Organization (WHO), the WHOQOL-BREF and the WHOQOL-OLD, and compared them to two control groups. The mean global QoL score was 60.1 in nursing homes, slightly higher than in a comparable home-living group (54.8; *P* = 0.085) but significantly lower than in the general population (72.4; *P* < 0.001). The highest rated domain was fear of death and dying (81.2), the lowest autonomy (56.3). Multivariate analyses showed that female sex and surviving cancer improved some WHOQOL-BREF domains, while older age, higher care level, diabetes, and stroke reduced certain WHOQOL-OLD domains. Overall, QoL in nursing homes was high, even higher than among home-living peers, but lower than in healthier, younger populations. Fear of the end of life was low; autonomy declined most. Stroke notably reduced QoL, whereas long-term cancer survival was linked to better outcomes.

## Introduction

For older people, quality of life (QoL)—particularly health-related QoL—is an essential health indicator and can also serve as a quality indicator for care [1]. This is especially true for institutions such as nursing homes, which serve as permanent residences for individuals, going beyond merely treating illness or conducting diagnoses, as is the case in hospitals for acute illnesses. In these settings, the parameters of QoL provide valuable insight into how satisfied the individuals are, how they feel, and how they live there. For this reason, QoL is frequently assessed systematically in nursing homes [2–4].

While QoL describes a person’s wellbeing and encompasses various dimensions, it is defined in a very subjective manner and therefore varies from person to person. It is shaped by personal values, experiences, and expectations. It is a multidimensional construct that explores an individual’s subjective wellbeing based on information about their physical and mental health and social environment, under consideration of demographic factors [5].

The World Health Organization (WHO) defines QoL as “*… an individual’s perception of their position in life in the context of the culture and value systems in which they live and in relation to their goals, expectations, standards and concerns*” [6]. In order to measure it and enable comparisons, the WHO has created tools that cover various aspects of QoL from different areas which have a decisive influence [7].

The dimensions of QoL, as suggested by the WHO, are physical, psychological, and social QoL, with environmental QoL often surveyed as a separate dimension. For older people, additional dimensions of QoL are taken into account, such as the sensory dimension, social participation, spirituality, coming to terms with the past and future perspectives, the feeling of autonomy, dealing with death and dying, and intimacy [8]. The QoL of older people is influenced by many different factors. In addition to health-related factors, these include demographic factors such as age, gender, education, and occupation, which are usually directly related to socioeconomic status [9]. In nursing homes, in addition to the physical and psychological dimensions of QoL that are mostly assessed, older-specific dimensions like autonomy, participation, and sensory abilities are less frequently evaluated, but are still very important [10].

The systematic assessment of QoL in nursing homes is crucial for gaining a realistic understanding of how residents are actually doing. Many of them are unable to express themselves due to health-related limitations or may fear negative consequences if they report problems. From the perspective of the care facility, assessing QoL serves as a tool for quality assurance, and the analysis has therefore become an aim of quality management [4, 11]. Beyond data collection itself, comparing the results is particularly important—without a benchmark, it is difficult to determine whether the outcomes are satisfactory or require improvement.

The aim of this study was to survey the QoL of institutionalized people in geriatric care homes of the Haus der Barmherzigkeit, to compare this with frail or prefrail older people living at home and the general population aged 65 years and older, and to analyze which health parameters are associated with QoL.

## Methods

### Study participants

The Haus der Barmherzigkeit, with its seven geriatric care homes in Vienna and Lower Austria, is, apart from state-run facilities, one of the largest private not-for-profit care and nursing institutions for people with chronic illnesses in Austria. These facilities primarily provide care for older individuals with high levels of care needs.

For this study, Stephansheim in Horn, Stadtheim in Wiener Neustadt (both located in predominantly rural Lower Austria), and the nursing home Am Maurer Berg in the city of Vienna were selected. At Stephansheim, approximately 143 residents are cared for, including those in hospice care, short-term care, and transitional care. Stadtheim comprises 162 beds, including short-term and transitional care. Up to 65 residents are cared for at Am Maurer Berg, also including short-term care. If needed, medical doctors as well as therapists are available in the immediate vicinity to care for the residents, although they are not employed by Haus der Barmherzigkeit.

Participants included in the study were all residents of the Stephansheim, Stadtheim, and Am Maurer Berg nursing homes who were receiving long-term geriatric care as of the respective reference dates (13 November 2023 at Am Maurer Berg; 23 September 2024 at Stadtheim; 24 October 2024 at Stephansheim). Residents with medical or cognitive impairments so severe that they were unable to participate in an interview were excluded from the study. In this context, medical impairments refer to physical limitations that could significantly affect communication or the wellbeing of interview participants during the conversation. Cognitive impairments are conditions that reduce participants’ mental abilities. These include the inability to understand complex contexts, concentration difficulties, confusion, disorientation, and impaired judgement. Another important exclusion criterion was the absence of consent to participate in the study, either from the individual or their legal representative.

### Control cohorts

The Healthy for Life cohort derives from a study conducted with older people who participated in a randomized controlled trial of a 12-week nutritional and physical activity intervention program, carried out between September 2013 and July 2015 in Vienna, Austria. Participants were included if they were aged 65 years or older; still living in their own homes; able to walk; and either at risk of malnutrition, malnourished, prefrail, or frail [12]. Baseline variables from 80 participants of the Healthy for Life cohort with complete QoL data were included in our analysis.

This study also considers the participants of the 2019 Austrian Health Interview Survey (ATHIS) [13], a nationwide survey designed to collect information on the population’s health status, health determinants, and socioeconomic background in private households across Austria. The target population comprised individuals aged 15 years and older registered in Austria’s national central population register. For our study, we extracted data from participants aged 65 and older grouped into two regions—Vienna and Lower Austria (the two federal Austrian states in which the included nursing homes are located)—resulting in 322 and 625 participants, respectively. The survey was conducted from October 2018 to September 2019. While ATHIS was principally conducted via computer-assisted personal interviews (CAPI) and web-based interviews (CAWI), questions regarding QoL were collected through a self-administered questionnaire, either in computer-based or paper–pencil format [13].

### Measurements

Many instruments have been developed to measure QoL in institutionalized older adults, each with advantages and limitations. The instruments developed by the WHO are intended for cognitively intact residents and are widely used [14]. This study used the WHOQOL-BREF, a standardized questionnaire developed by the WHO in 1998 to analyze QoL. The WHOQOL-BREF is a shortened version of the WHOQOL-100, which assesses a total of 100 items related to QoL [7]. Information on physical and mental health, social relationships, and the environment of interview participants is collected using 26 items. The questions are answered on a Likert scale ranging from 1 to 5. The WHOQOL-BREF is used in many countries and allows international comparison of the QoL of people in different cultures [15]. The WHOQOL-BREF was used in all three cohorts of the study.

In the nursing homes of Haus der Barmherzigkeit, and partly in the Healthy for Life cohort, the WHOQOL-OLD tool was also applied. This is an additional module comprising six domains, each divided into four subitems. The WHOQOL-OLD examines sensory abilities; perception of one’s autonomy; past, present, and future activities; social participation; intimacy; and personal confrontation with death and dying [8].

The aim of both the WHOQOL-BREF and the WHOQOL-OLD is not only to assess needs and satisfaction, but also to provide institutions with reference points for quality improvement, as well as to identify weaknesses in the healthcare and care systems [6]. A score ranging from 0 to 100 was calculated for all domains of the WHOQOL-BREF and WHOQOL-OLD, with 0 representing the lowest QoL and 100 the highest.

Other variables included in the study were medical diagnoses, either retrieved from the respective documentation systems or reported by the participants via the questionnaires used. These were classified according to the categories used in the ATHIS survey to enable comparability. Only diagnoses present in all cohorts were analyzed and compared.

Additionally, data on body height and weight (self-reported, measured directly, or estimated using knee–heel length) were collected, and body mass index (BMI) was calculated in kg/m^2^ and categorized into underweight, normal weight, overweight, and obesity, in line with WHO classifications [16]. Furthermore, the level of care (*Pflegestufe*), a tax-based cash benefit indicating the degree of nursing care required [17], was recorded. This measure reflects how restricted an individual is in their independence [18]. Finally, the highest level of education completed, sex, and age were either asked directly or taken from electronic routine documentation and used as variables.

### Statistics

The following statistical methods were applied: descriptive statistics were used, with frequencies (in percentages) reported for categorical variables. For normally distributed metric variables, the mean was reported. To test for differences between groups, the chi^2^ test was used for categorical variables, and either a t-test or analysis of variance (ANOVA) was applied for metric variables to compare means. Additionally, linear regressions were calculated with the respective QoL domains as the dependent variables. Independent variables included age, sex, level of care, and medical diagnoses. To exclude multicollinearity, correlation analyses were conducted in advance using these variables. Through the regression analysis and standardization, variables could be compared with one another. The results of linear regression are presented as standardized beta coefficients. Beta values represent the positive or negative impact of each predictor on the respective QoL domains. The model fit was assessed using the R^2^ value.

The QoL assessments in the Haus der Barmherzigkeit nursing homes were discussed and approved by the Ethics Commission of Haus der Barmherzigkeit on 13 November 2023 for the Vienna nursing home and on 6 June 2024 for the nursing homes in Lower Austria. The Healthy for Life study was approved by the Ethics Committee of the Medical University of Vienna (ref: EK1416/2013) and the Ethics Committee of the City of Vienna (ref: EK13-240-1113). The secondary analysis of the ATHIS database was approved by the Ethics Committee of the Medical University of Vienna (ref: EK1263/2021).

## Results

On the respective reference dates, 334 residents were living in long-term geriatric care at the Haus der Barmherzigkeit nursing homes; 62 individuals were not included due to health-related reasons preventing participation, and a further 55 individuals were excluded because either they or their legal representatives declined to participate. Of the remaining 217 residents, 190 had valid QoL data available for analysis. Thus, the response rate was 56.9% (52.3% at Am Maurer Berg, 58.5% at Stephansheim in Horn, and 57.5% at Stadtheim in Wiener Neustadt).

Table 1 shows the key characteristics of all analyzed populations: residents of the nursing homes (Am Maurer Berg, Stephansheim, and Stadtheim), (pre)frail individuals living at home in Vienna within the Healthy for Life cohort, and the general population aged 65 and older in Vienna and Lower Austria. The proportion of women was significantly higher in the nursing homes and in the Healthy for Life study as compared to the general population. The mean age in the nursing homes and in the Healthy for Life cohort was around 10 years higher than in the general population.

**Table 1:** Sociodemographic and health parameters in the included populations

	AMB *N* = 34	HOR *N* = 72	WRN *N* = 84	HFL *N* = 80	AT‑V *N* = 322	AT-LA *N* = 625	*P*-value^a^
*Sex (%)*							
Male	35.3	25.0	19.0	16.3	46.9	46.4	< 0.001
Female	64.7	75.0	81.0	83.8	53.1	53.6	
*Age, years (mean)*	85.4	83.9	85.8	82.8	75.1	75.6	< 0.001
*Education (%)*							
Primary or apprenticeship	85.3	94.9	94.9	53.8	55.6	84.8	< 0.001
Secondary	8.8	5.1	5.1	33.8	27.3	9.0	< 0.001
Tertiary	5.9	0.0	0.0	12.5	17.1	6.2	< 0.001
*Body mass index category (%)*							
Underweight	3.2	2.8	7.1	1.3	0.3	0.8	< 0.001
Normal weight	32.3	34.7	48.8	30.0	34.2	30.2	0.031
Overweight	38.7	25.0	15.5	47.5	40.7	46.7	< 0.001
Obesity	25.8	37.5	26.8	21.3	24.8	22.2	0.088
*Diagnoses (%)*							
Diabetes mellitus	17.6	30.6	28.6	20.0	18.3	18.4	0.060
Hypertension	73.5	70.8	61.9	75.0	51.6	52.6	< 0.001
CHD	23.5	23.6	20.2	7.5	12.7	13.9	0.019
History of stroke	29.4	25.0	19.0	8.8	5.0	7.4	< 0.001
COPD or asthma	17.6	4.1	13.1	16.3	13.0	11.4	0.203
Cancer	26.5	20.8	21.4	8.8	7.5	5.9	< 0.001
Depression	47.1	30.6	32.1	10.0	10.6	9.3	< 0.001

*AMB* nursing home Am Maurer Berg in Vienna; *HOR* nursing home Stephansheim in Horn, Lower Austria; *WRN* nursing home Stadtheim in Wr. Neustadt, Lower Austria; *HFL* Healthy for Life cohort in Vienna; *AT‑V* ATHIS-19 sample aged 65+ in Vienna; *AT-LA* ATHIS-19 sample aged 65+ in Lower Austria; *CHD* coronary heart disease; *COPD* chronic obstructive pulmonary disease

^a^*P*-value as result of chi^2^ test for categorial parameters and analysis of variance (ANOVA) for mean of metric variables

Significant differences were observed in terms of the highest level of education completed. Significantly more people with lower educational attainment were found in the Lower Austrian study populations than in the Viennese populations. Moreover, educational attainment was also lower on average in the nursing homes compared to the home-living populations. Regarding the BMI, there were significantly more individuals who were underweight or obese in the nursing homes compared to those living at home. Clear differences were also evident in terms of medical diagnoses. Almost all diagnoses were more prevalent in the nursing home populations than in the Healthy for Life cohort and more prevalent in the Healthy for Life cohort than in the general population. Notably, the diagnoses of coronary heart disease, stroke, cancer, and depression were particularly common in the nursing home cohorts.

The mean score for global QoL in the Haus der Barmherzigkeit nursing homes was 60.1. This was slightly higher than the value for the Healthy for Life cohort (54.8; *P* = 0.085) and significantly lower than the mean in the ATHIS database (72.4; *P* < 0.001). For the domains of physical health, psychological health, social relationships, and environment, the mean scores in the Haus der Barmherzigkeit nursing homes were 58.1, 64.0, 63.6, and 72.3, respectively. All scores except for those in the environment domain were higher than the respective means in the Healthy for Life cohort (48.6, *P* < 0.001; 61.0, *P* = 0.207; 45.1, *P* < 0.001; and 75.3, *P* = 0.154; respectively). Mean values for all QoL domains in the Haus der Barmherzigkeit nursing homes were significantly lower (*P* < 0.001 for all comparisons) than those in the ATHIS database (70.7, 76.9, 73.0, and 79.5, respectively). The values for all three nursing homes, the Healthy for Life cohort, and the general population in the two Austrian federal states are shown in Table 2.

**Table 2:** Mean values for different domains in quality of life (WHOQOL-BREF) in included populations

	AMB *N* = 34	HOR *N* = 72	WRN *N* = 84	HFL *N* = 80	AT‑V *N* = 322	AT-LA *N* = 625	*P*-value^a^
Global value	62.1	59.8	59.5	54.8	73.1	72.0	< 0.001
Physical health	57.7	60.0	56.8	48.6	71.8	70.1	< 0.001
Psychological health	69.3	59.5	65.6	61.0	78.2	76.2	< 0.001
Social relationships	60.3	62.6	65.8	45.1	72.4	73.3	< 0.001
Environment	73.7	72.5	71.4	75.3	80.9	78.9	< 0.001

*AMB* nursing home Am Maurer Berg in Vienna; *HOR* nursing home Stephansheim in Horn, Lower Austria; *WRN* nursing home Stadtheim in Wr. Neustadt, Lower Austria; *HFL* Healthy for Life cohort in Vienna; *AT‑V* ATHIS-19 sample aged 65+ in Vienna; *AT-LA* ATHIS-19 sample aged 65+ in Lower Austria

^a^*P*-value as result of analysis of variance (ANOVA) for mean of metric variables

There were no statistically significant differences between the mean values of the WHOQOL-OLD domains between the Haus der Barmherzigkeit nursing homes and the Healthy for Life cohort in the domains of sensory abilities (64.9 vs. 60.9; *P* = 0.266); past, present, and future activities (61.5 vs. 64.8; *P* = 0.256); and social participation (60.2 vs. 55.6; *P* = 0.073). However, QoL in the autonomy domain was significantly lower in the Haus der Barmherzigkeit homes compared to the Healthy for Life cohort (56.3 vs. 67.2; *P* < 0.001). The WHOQOL-OLD total score in the nursing homes was 64.2, whereby the score for death and dying was 81.2 and for intimacy 60.8. Table 3 shows the respective scores for all three nursing homes and for the Healthy for Life cohort (where available).

**Table 3:** Mean values for different domains in quality of life (WHOQOL-OLD) in included populations

	AMB *N* = 34	HOR *N* = 72	WRN *N* = 84	HFL *N* = 80	*P*-value^a^
WHOQOL-OLD total	63.1	61.4	67.1	–	0.089
Sensory abilities	63.6	64.0	66.2	60.9	0.669
Autonomy	54.3	50.1	62.0	67.2	< 0.001
Past, present, and future activities	58.7	55.2	67.5	64.8	0.003
Social participation	52.4	58.1	65.3	55.6	0.007
Death and dying	83.9	82.7	78.7	–	0.471
Intimacy	65.4	58.2	60.8	–	0.374

*AMB* nursing home Am Maurer Berg in Vienna; *HOR* nursing home Stephansheim in Horn, Lower Austria; *WRN* nursing home Stadtheim in Wr. Neustadt, Lower Austria; *HFL* Healthy for Life cohort in Vienna

^a^*P*-value as result of analysis of variance (ANOVA) for mean of metric variables

Sociodemographic variables such as age and the level of care were not significantly associated with any of the WHOQOL-BREF domains in the multivariate analysis. Sex was significantly associated with the domain of social relationships. None of the health-related variables (diabetes mellitus, hypertension, coronary heart disease, history of stroke, chronic obstructive pulmonary disease or asthma, or depression) were significantly associated with any WHOQOL-BREF domain. Cancer, however, was significantly associated with psychological health in the multivariate model (Table 4).

**Table 4:** Sociodemographic and health-related variables influencing quality of life (WHOQOL-BREF) in nursing homes of Haus der Barmherzigkeit

	Global value	Physical health	Psychological health	Social relations	Environment
Age	0.114	−0.005	0.016	0.110	0.013
Sex	0.65	−0.015	0.081	**0.219	0.164
Level of care	−0.001	−0.090	−0.106	−0.018	−0.088
Diabetes mellitus	0.007	−0.116	−0.050	0.064	0.099
Hypertension	0.026	0.073	0.020	0.017	0.001
CHD	0.054	0.032	0.043	0.024	0.084
History of stroke	−0.055	−0.146	−0.113	−0.096	−0.027
COPD or asthma	−0.110	−0.058	0.096	−0.054	0.042
Cancer	0.012	0.135	**0.196	0.065	0.100
Depression	−0.029	−0.087	−0.062	0.020	0.012
R^2^	0.041	0.069	0.082	0.100	0.067

Results of multiple linear regression analysis shown as standardized beta coefficients representing the positive (coefficients above 0) and negative (coefficients below 0) associations between each predictor and the respective quality of life domain

*CHD* coronary heart disease, *COPD* chronic obstructive pulmonary disease

**P < 0.01

Regarding influences on the domains of the WHOQOL-OLD (Table 5), age and level of care were significantly associated with certain domains: age was associated with sensory abilities, and level of care with the domain of autonomy. Diabetes mellitus was significantly associated with fears about death and dying, and having had a stroke was associated with reduced social participation.

**Table 5:** Sociodemographic and health-related variables influencing quality of life (WHOQOL-OLD) in nursing homes of Haus der Barmherzigkeit

	Total	Sensory abilities	Autonomy	Past, present, and future activities	Social participation	Death and dying	Intimacy
Age	−0.055	−0.351***	−0.014	0.120	0.088	−0.019	0.021
Sex	0.089	0.125	0.061	0.084	0.059	−0.111	0.110
Level of care	−0.151	−0.118	−0.164*	−0.127	−0.101	−0.039	−0.006
Diabetes mellitus	−0.096	−0.108	−0.021	−0.012	−0.013	−0.197*	−0.005
Hypertension	−0.004	0.038	0.029	0.043	−0.017	−0.066	0.046
CHD	0.066	0.011	0.063	0.009	−0.012	0.090	0.104
History of stroke	−0.152	−0.109	−0.120	−0.110	−0.195*	−0.096	−0.009
COPD or asthma	0.004	0.043	0.044	−0.041	−0.018	−0.018	0.024
Cancer	0.090	0.127	0.016	0.149	0.001	0.026	0.043
Depression	−0.042	−0.106	−0.034	−0.029	0.013	−0.039	0.020
R^2^	0.082	0.148	0.057	0.093	0.077	0.068	0.034

Results of multiple linear regression analysis shown as standardized beta coefficients, representing the positive (coefficients above 0) and negative (coefficients below 0) associations between each predictor and the respective quality of life domain.

*CHD* coronary heart disease, *COPD* chronic obstructive pulmonary disease

*P < 0.05, **P < 0.01, ***P < 0.001

## Discussion

Overall, the findings of this study indicate that the quality of life (QoL) in the Haus der Barmherzigkeit nursing homes is quite high. It was higher compared to the Healthy for Life cohort, except for in the domains of environment and autonomy, despite a higher prevalence of chronic diseases, particularly ischemic heart disease, stroke, cancer, and depression. However, the QoL in nursing homes did not reach the level observed in the general older population. This may be attributed to the fact that the ATHIS-19 participants were significantly younger, more highly educated, and demonstrated a higher health status than the nursing home residents, especially regarding ischemic heart disease, stroke, cancer, and depression.

A similar result was found in a meta-analysis comparing the QoL of individuals living in nursing homes with those living at home and cared for by family members. In this analysis, QoL was also reported to be higher among those living at home [19]. Institutionalization itself appears to have an impact on QoL, as suggested by another meta-analysis, which, beside other measures, used both the WHOQOL-BREF and WHOQOL-OLD instruments that we also used in our study [9].

The domain that clearly showed the lowest QoL values in the nursing homes was autonomy. Often, residents have to leave their homes and familiar surroundings behind to move into a nursing home. As a result, they may feel limited in their decision-making processes. This, and the general decline in health and increase in dependency, often leads to a loss of autonomy. Autonomy, alongside other factors such as leisure activities, religion, spirituality, sexuality, and end-of-life care wishes, is one of the most frequently articulated needs and wishes of nursing home residents that defines their QoL [20].

The QoL values in the nursing homes were highest in the domain of concerns and fears about death and dying, indicating that participants rated their QoL in this domain higher than in any other domain of both the WHOQOL-OLD and the WHOQOL-BREF. This could be explained by the spiritual support provided in the Haus der Barmherzigkeit nursing homes, which have a confessional foundation. Indeed, spirituality has been shown to be a key domain that defines QoL in older adults [21]. Additionally, the nursing homes offer advanced care planning, which includes precautionary conversations with residents and their families, as well as discussions with the residents’ primary caregiver and physician. These conversations address the residents’ wishes for a meaningful life in the nursing home as well as their preferences when death becomes foreseeable. Such discussions may contribute to higher scores in this domain and help alleviate the fear of death and dying. Research has shown that advance care planning interventions in nursing homes are positively associated with several outcomes, including process improvements, quality of care, and health-related outcomes such as QoL [22]. Finally, the constant presence of nursing staff, the availability of medical professionals, and access to palliative care in the nursing homes may further reduce concerns that dying could be associated with pain or suffering.

The age of the residents only influenced QoL in the domain of sensory abilities, not in any other domain. Their responses reflected a decline in satisfaction regarding eyesight, hearing, and other senses. Analyzing the specific sensory impairments and their impact on particular activities (e.g., the influence of declining eyesight on reading) could be an interesting avenue for further research, particularly in relation to cognitive decline, as it too negatively affects QoL [23].

Sex had an influence only on the social relations domain, where women rated their QoL higher than men. Previous studies found that men in nursing homes tend to be less satisfied with activities, have fewer friends, and rely less on their families for support, which often results in a lower QoL across several domains [24]. Additionally, research using data from the Survey of Health, Ageing and Retirement in Europe found that men who had lost a partner became and remained frail, whereas women gradually recovered from partner loss [25]. This disparity could potentially be attributed to women’s better social connections and their greater use of social support as a coping strategy during adverse life events.

In our study, the level of care was associated with a decrease in QoL in the autonomy domain. One possible reason for this could be that individuals with a higher level of care may be more bedridden and thus more restricted in movement, perceiving this as a loss of autonomy compared to those with a lower level of care. An interesting follow-up question for future research would be why the level of care, and consequently being bedridden, did not influence the physical domain of QoL.

The results regarding the different medical diagnoses revealed interesting associations with QoL. For instance, having had a stroke negatively affected QoL in the domain of social participation. This could be due to conditions such as sleep attacks, paralysis, speech impairments, and sensory function losses, which significantly hinder social interaction [26]. On the other hand, having a cancer diagnosis seemed to improve certain aspects of QoL, especially the psychological health domain. This contrasts with pooled findings from a meta-analysis, in which cancer survivors generally had worse QoL, although this was not consistent across all studies included [27]. In a study involving long-term cancer survivors, the QoL was found to exceed age-related norms [28]. A possible explanation for this could be the “survivor effect” of these residents, where surviving a serious illness results in an enhanced appreciation of life.

The study highlights the importance of an interprofessional approach in nursing homes, particularly to addressing autonomy and chronic conditions that significantly impact QoL. By involving inhabitants and their relatives, together with a range of healthcare professionals like nurses, doctors, social workers, and therapists, care plans can be tailored to respect individual preferences, promote autonomy, and enhance wellbeing. In the long term, this study suggests a need for continuous QoL assessments and the integration of tailored care models that adapt to residents’ evolving needs. Regular evaluations of various QoL domains as part of a routine comprehensive geriatric assessment can guide interventions and prevent declines in wellbeing [29].

A strength of this study is the inclusion of three nursing homes, yielding relatively consistent results, and the comparability of these results with two different cohorts: one that was similar to the nursing home residents in terms of age and health but who lived at home, and the older general population from the two federal states in which the nursing homes were located. The simultaneous collection of health data (including diagnoses of chronic diseases) and sociodemographic factors also constitutes a strength. Additionally, the use of frequently used and well-validated survey instruments (WHOQOL-BREF and WHOQOL-OLD) adds robustness to the study. Another strength is the goal of surveying all nursing home residents in long-term geriatric care, aiming to include the entire population of institutionalized older adults within the selected facilities. However, this is also the study’s strongest limitation, as fewer than two thirds of the residents could be included. Many individuals were excluded due to health or cognitive factors or due to a lack of consent, potentially skewing the results toward an overly positive representation of QoL. Moreover, the questionnaires were sometimes perceived as difficult to administer, with certain questions, such as those regarding satisfaction with work ability or sexual life, being seen as inappropriate by many residents. The questionnaires were also considered too long in many cases, necessitating multiple breaks during the survey.

Comparing QoL outcomes across cohorts with different data collection periods ranging from 2013–2015 (Healthy for Life) to 2023–2024 (Haus der Barmherzigkeit nursing homes) also presents certain limitations. The observed QoL differences may, at least in part, reflect external influences that are unrelated to the participants themselves, such as climatic or economic conditions. Nevertheless, the comparisons serve as a useful starting point for exploring QoL differences within the diverse population of older adults in Austria.

## Conclusion

The quality of life (QoL) of residents was systematically assessed through a standardized survey, ensuring both valid evaluations and comparability across different care facilities and similar cohorts. This methodology provides a robust foundation for further research and offers valuable insights for professionals and decision-makers, highlighting areas for targeted quality improvement. The in-depth examination of individual QoL domains enables identification of specific measures that can be implemented within an interdisciplinary setting to enhance the living conditions and wellbeing of residents on a personalized basis.

The findings of the study indicate that overall, QoL in nursing homes is very high, with some aspects even surpassing those of a comparison group of individuals living at home with similar social and health characteristics. However, QoL still remains lower than that of the younger, healthier general population. Notably, while concerns about death were not markedly pronounced, the most significant decline in QoL was observed in the domain of autonomy. Certain medical conditions, especially stroke, were found to negatively impact QoL, while long-term cancer survivors displayed exceptionally high QoL scores. These findings suggest that the greatest potential for improvement in nursing home care lies in addressing issues related to autonomy, and further interventions may be needed to support residents with chronic conditions, particularly those recovering from strokes.
